# Linc-RoR promotes MAPK/ERK signaling and confers estrogen-independent growth of breast cancer

**DOI:** 10.1186/s12943-017-0727-3

**Published:** 2017-10-17

**Authors:** Wan-xin Peng, Jian-guo Huang, Liu Yang, Ai-hua Gong, Yin-Yuan Mo

**Affiliations:** 1 Department of Cell biology, School of Medicine, Jiangsu University, Zhenjiang, China; 2 Cancer Institute, University of Mississippi Medical Center, Jackson, MS USA; 3 Department of Biochemistry, University of Mississippi Medical Center, Jackson, MS USA; 4 Department of Science & Research, Zhejiang Provincial People’s Hospital, Hangzhou, China; 5 Department of Pharmacology/Toxicology, University of Mississippi Medical Center, Jackson, MS USA

**Keywords:** Breast cancer, Estrogen-independent growth, Linc-RoR, ERK, DUSP7

## Abstract

**Background:**

The conversion from estrogen-dependent to estrogen-independent state of ER+ breast cancer cells is the key step to promote resistance to endocrine therapies. Although the crucial role of MAPK/ERK signaling pathway in estrogen-independent breast cancer cell growth is well established, the underlying mechanism is not fully understood.

**Methods:**

In this study, we profiled lncRNA expression against a focused group of lncRNAs selected from lncRNA database. CRISPR/Cas9 was employed to knockout (KO) linc-RoR in MCF-7 cells, while rescue experiments were carried out to re-express linc-RoR in KO cells. Colony formation and MTT assays were used to examine the role of linc-RoR in estrogen-independent growth and tamoxifen resistance. Western blot and qRT-PCR were used to determine the change of protein and lncRNA levels, respectively. The expression of DUSP7 in clinical specimens was downloaded from Oncomine (www.oncomine.org) and the dataset from Kaplan-Meier Plotter (http://kmplot.com) was used to analyze the clinical outcomes in relation to DUSP7.

**Results:**

We identified that linc-RoR functions as an onco-lncRNA to promote estrogen-independent growth of ER+ breast cancer. Under estrogen deprivation, linc-RoR causes the upregulation of phosphorylated MAPK/ERK pathway which in turn activates ER signaling. Knockout of linc-RoR abrogates estrogen deprivation-induced ERK activation as well as ER phosphorylation, whereas re-expression of linc-RoR restores all above phenotypes. Moreover, we show that the ERK-specific phosphatase Dual Specificity Phosphatase 7 (DUSP7), also known as MKP-X, is involved in linc-RoR KO-induced repression of MAPK/ERK signaling. Interestingly, linc-RoR KO increases the protein stability of DUSP7, resulting in repression of ERK phosphorylation. Clinical data analysis reveal that DUSP7 expression is lower in ER+ breast cancer samples than that in ER- breast cancer. Moreover, downregulation of DUSP7 expression is associated with poor patient survival.

**Conclusion:**

Taken together, these results suggest that linc-RoR promotes estrogen-independent growth and activation of MAPK/ERK pathway of breast cancer cells by regulating the ERK-specific phosphatase DUSP7. Thus, this study might help not only in establishing a role for linc-RoR in estrogen-independent and tamoxifen resistance of ER+ breast cancer, but also suggesting a link between linc-RoR and MAPK/ERK pathway.

**Electronic supplementary material:**

The online version of this article (10.1186/s12943-017-0727-3) contains supplementary material, which is available to authorized users.

## Background

Approximately 70–80% of breast cancers express estrogen receptor alpha (hereafter referred to as ER) and they depend on estrogen signals for continued growth [1]. Hence, endocrine therapies targeting ER using tamoxifen or aromatase inhibitors (AIs) are the first-line adjuvant therapies offered to patients with ER-positive (+) breast cancer [2]. However, whatever the hormonal strategies are used, intrinsic or acquired resistance may occur [3, 4]. Increasing evidence indicates that estrogen-independent growth develops in a significant proportion of patients, leading to hormonal therapy resistance. Several molecular mediators of estrogen-independent growth have been described, such as regulation of microRNAs [5], growth factor signaling crosstalk [6, 7], and mutation of ER [8].

Long non-coding RNAs (lncRNAs) are currently defined group of transcripts that are more than 200 nucleotides in length. Despite lacking of the protein-coding potential, emerging evidence indicates that lncRNAs may serve as master gene regulators through various mechanisms, and thus, the dysregulation of lncRNAs expression is often linked to diverse human diseases including cancer [9, 10]. Although we are only beginning to understand the function of lncRNAs, increasingly evidence have led the belief that lncRNAs may regulate gene expression through interacting with DNA, RNA, or protein. Recent studies have implicated some lncRNAs in endocrine resistance in breast cancer [11]. For instance, a recent report showed that the lncRNA HOTAIR is upregulated in tamoxifen-resistant ER+ breast cancers and thus contributed to tamoxifen resistance [12]. More recently, studies from other groups suggest that lncRNA UCA1 confers tamoxifen resistance through different signaling pathways [13, 14]. However, the underlying mechanism of how lncRNAs regulate intrinsic hormonal resistance and estrogen-independent growth is still far from being understood.

LincRNA regulator of reprogramming (linc-RoR) was originally identified to be able to regulate reprogramming of iPSCs [15]. A subsequent report demonstrates that linc-RoR may serve as microRNA sponge to regulate the expression of the core transcription factors Oct4, Sox2, and Nanog in human embryonic stem cell self-renewal [16]. Our previous study reports that linc-RoR inhibits p53 translation by interacting with heterogeneous nuclear ribonucleoprotein I (hnRNP I) in response to DNA damage [17], and further studies suggest a link between linc-RoR and oncogene c-Myc in cancer progression [18]. Of interest, it is reported that linc-RoR is dramatically overexpressed in triple negative breast cancer (TNBC), and the expression of linc-RoR promotes cell invasion via miR145/ARF6 pathway [19]. However, little is known as to whether linc-RoR have any role in estrogen-independent growth of ER+ tumors.

The present study demonstrates that linc-RoR functions as a modulator to promote estrogen-independent growth and tamoxifen resistance of breast cancer cells. Linc-RoR expression is upregulated in response to estrogen deprivation. While knockout of linc-RoR abolishes estrogen-independent growth of MCF-7 cells, rescue assays reverse the phenotype. Importantly, linc-RoR confers MAPK/ERK pathway activation by decreasing the stability of phosphatase DUSP7 which is the negative regulator of ERK, suggesting a novel role for linc-RoR in regulation of MAPK/ERK signaling in response to estrogen deprivation.

## Methods

### Reagents

Sources of primary antibodies were: pERα (16 J4), ERα (D6R2W), pERK1/2 (D13.14.4E), ERK1/2(137F5), pMEK1/2(41G9), MEK1/2(D1A5) and MKP-2 (D9A5) from Cell Signaling (Danvers, MA, USA); MKP-1(D-3) and MKP-3 (F-12) from Santa Cruz (Dallas, TX, USA); DUSP7/MKP-X(26910–1-AP) and GAPDH (60004–1-Ig) from Proteintech (Rosemont, IL, USA). Secondary antibodies conjugated with IRDye 800CW or IRDye 680 were purchased from LI-COR Biosciences (Lincoln, NE, USA). PCR primers were purchased from IDT (Coralville, IA, USA). U0126 and NSC95397 were purchased from Cell Signaling and Santa Cruz (Dallas, TX, USA), respectively. TAM was obtained from Sigma-Aldrich (St. Louis, MO, USA).

### Cell culture

MCF-7 parental, gRNA control and RoR KO cells were maintained in RPMI 1640 medium supplemented with 10% FCS. For estrogen deprivation treatment, these cells were cultured in phenol red-free RPMI medium containing 5% charcoal stripped FCS (E2-free medium). All the medium contain 2 mM L-glutamine, 100 U/ml penicillin and 100 μg/ml streptomycin.

### LncRNA profiling

For lncRNA profiling, we used Human Disease-Related LncRNA Profiler, as described previously [20]. Total RNA was extracted using Direct-zol™ RNA MiniPrep (Zymo Research) and cDNA synthesis was carried out using RevertAid™ Reverse Transcriptase (Thermo Fisher) with random primers. Delta-delta Ct values (regular medium versus E2-free medium) were used to determine their relative expression as fold changes, as previously described [21].

### Western blot

Cells were harvested, and proteins were extracted and quantified as previously described [5]. Protein samples were separated in a polyacrylamide SDS gel before transferring to PVDF membrane. After probing with a primary antibody, the membrane was incubated with a secondary antibody labeled with either IRDye 800CW or IRDye 680. Finally, signal intensity was detected by the Odyssey Infrared Imaging System (LI-COR Biosciences, Lincoln, NE, USA). The intensities of band were quantified by imageJ software (version 1.6.0, Windows, NIH).

### Quantitative RT-PCR (qRT-PCR)

PCR was performed using a standard SYBR Green method (Bio-Rad, Hercules, CA, USA) as previously described [22]. We used primers linc-RoR-RT-5.1A and linc-RoR-RT-3.1A; pS2-RT-5.1 and pS2-RT-3.1; CXCL12-RT-5.1 and CXCL12-RT-3.1; c-Myc-RT-5.1 and c-Myc-RT-3.1; cyclin D1-RT-5.1 and cyclin D1-RT-3.1 to detect the mRNA level of these genes (Additional file 1: Table S1). GAPDH was used as an internal control. Delta-delta Ct values were used to determine their relative expression.

### KO of linc-RoR by CRISPR/Cas9

We used a dual gRNA approach to knock out exon 4 of linc-RoR (RoR E4) by CRISPR/Cas9 system [23, 24]. Briefly, dual gRNA and donor vector were co-transfected into MCF-7 cells. One week later, the transfected cells were subject to puromycin (1 μg/ml) selection; and surviving cells were sorted by FACS based on GFP signal into 96-well plates and then expanded in 12-well plates. Potential clones were further verified by genomic PCR and qRT-PCR.

### Cell proliferation assay

Cell proliferation assays were carried out by MTT (3-(4,5-dimethylthiazol-2-yl)-2,5-diphenyltetrazolium bromide) assays, as previously described [25].

### In silico data analysis

Correlations between breast cancer patient survival and DUSP7 expression (probe: 214793_at) was analyzed by KM plotter (http://kmplot.com). Auto select best cutoff was chosen in the analysis. A total of 3951 breast cancer samples were split in high and low groups according to the cutoff value, respectively. The hazard ratio with 95% confidence intervals and log rank *P* value was calculated and significance was set at *P* < 0.05. The expression of DUSP7 in breast cancer grouped by ER status was identified from TCGA databases in Oncomine (Compendia Biosciences, www.oncomine.org) using the analysis of “Dataset view” as previously reported [26].

### Statistical analysis

All statistical analyses were performed using the GraphPad Prism Version 6.0 program. The continuous variables are summarized as mean and standard error of mean (S.E.M.) unless stated. The two-sample t test was used to compare the mean of a continuous variable between two samples. The Satterthwaite’s t test was used for mean comparison when the variances in two samples were unequal. Association between two categorical variables was evaluated by using the Fisher’s exact test. Relapse-free survival (RFS) curves were calculated with the Kaplan-Meier method and were analyzed with the log-rank test. All *P* values were two-sided and P values <0.05 were considered as significant.

## Results

### Linc-RoR is induced by estrogen deprivation

**Fig. 1:**
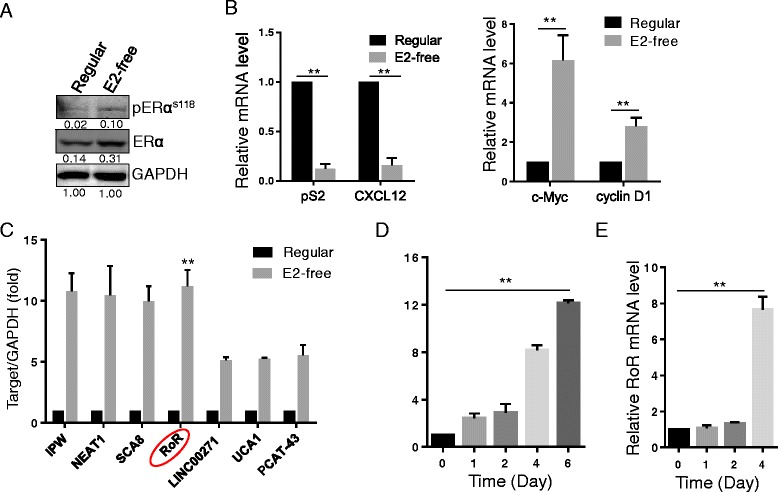
Identification of linc-RoR as an estrogen deprivation-induced lncRNA. **a** Detection of ER activation after 6 days E2-free treatment by Western blot. The intensity value is relative to GAPDH as 1. **b** Expression of ER genomic and non-genomic target genes after 6 days of E2-free treatment. **c** LncRNA profiling after 6 days of E2-free treatment. **d** Linc-RoR is induced by E2-free treatment in a time-dependent manner. **e** Induction of linc-RoR by TAM treatment. Error bars represent S.E.M., *n* = 3, **P < 0.01

Estrogen-independent growth of ER+ cells is one of important factors that leads to the failure of endocrine therapy [27]. To determine whether lncRNAs play a role in endocrine resistance, we asked whether any of lncRNAs is induced by estrogen deprivation. Therefore, we first treated MCF-7 cells with E2-free medium for 6 days, and then detected ERα activation at Ser118 by western blot (Fig. 1a). Meanwhile, we analyzed the expression of pS2, CXCL12 (two widely used genomic target of ERα); c-Myc and cyclin D1 (two widely used non-genomic target of ERα) by qRT-PCR (Fig. 1b). The statistically significant downregulation of pS2 and CXCL12 mRNA levels were observed after 6 days treatment with E2-free medium (Fig. 1b, left panel), while a dramatic upregulation was found in c-Myc and cyclinD1 mRNA level (Fig. 1b, right panel). These results suggest that ER signaling may be activated by non-genomic action mechanism and then modulating downstream target genes under 6 days E2-free condition. Therefore, we then chose E2-free culture for 6 days for profiling experiments and identified 7 lncRNAs with over a 5-fold of induction by estrogen deprivation (Fig. 1c). We were particularly interested in linc-RoR because a previous report suggests its potential oncogenic role in metastasis TNBC [19].

To further confirm the effect of estrogen deprivation on linc-RoR expression, we treated MCF-7 cells for different time points in E2-free medium (1, 2, 4 and 6 days) and then determined linc-RoR mRNA level by qRT-PCR. Linc-RoR expression was significantly induced by estrogen deprivation in a time-dependent manner (Fig. 1d). In addition, we also examined linc-RoR expression in response to E2 stimulation. The significant increase of linc-RoR was detected in 24 h E2-free treatment group but not in E2-free and E2 combined group (Additional file 1: Figure S1A), suggesting the negative regulation of linc-RoR by estrogen signal. Next, we asked whether linc-RoR expression is affected by tamoxifen (TAM). After MCF-7 cells were treated with 4 μM TAM for 1, 2 and 4 days, we detected ~8-fold increase in linc-RoR level at day 4 day, similar to the induction by estrogen deprivation (Fig. 1e). Collectively, the above results suggest that linc-RoR is important to estrogen-independent growth of MCF-7 cells. Therefore, linc-RoR was selected for further characterization of its role in estrogen-independent growth and tamoxifen resistance of breast cancer.

### Linc-RoR promotes estrogen independency and tamoxifen resistance

**Fig. 2:**
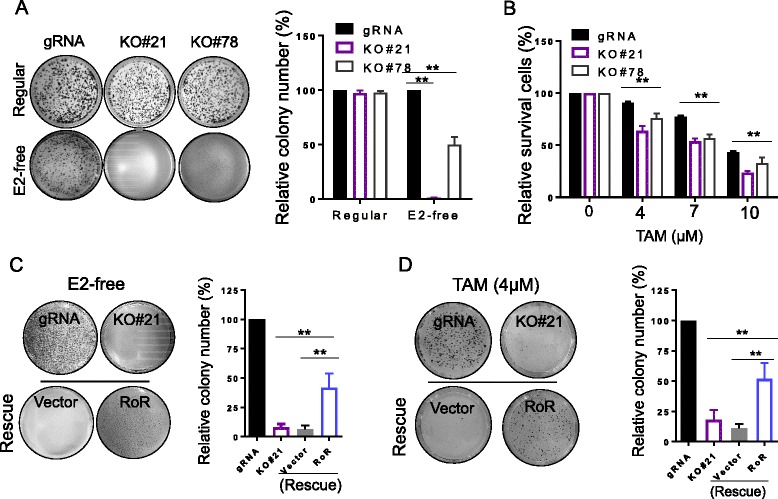
Linc-RoR KO suppresses estrogen-independent growth. **a** Linc-RoR KO suppresses estrogen-independent growth ad determined by clonogenic assays. **b** Linc-RoR KO sensitizes MCF-7 cells to TAM, as determined by MTT assay. **c** Re-expression of lincRoR partially restores the growth suppression by estrogen deprivation in linc-RoR KO cells. **d** Re-expression of lincRoR partially restores the TAM resistance in linc-RoR KO cells. Error bars represent S.E.M., n = 3, **P < 0.01

To determine the significance of linc-RoR in estrogen-independent growth, we took advantage of CRISPR/Cas9 system [23, 24] to knockout (KO) linc-RoR in MCF-7 cells since RNAi was not effective. Colony formation assays revealed that linc-RoR KO suppressed cell proliferation in E2-free condition (Fig. 2a, bottom left). Of interest, the level of linc-RoR was correlated with the colony number. For instance, there was little growth in KO#21. On the other hand, there was about 50% colony formation for KO#78. To our surprise, there was no difference in colony formation between vector control and linc-RoR KO in regular medium (Fig. 2a, up left), suggesting a role of linc-RoR in estrogen deprivation. Furthermore, MTT assays revealed that linc-RoR KO significantly enhanced the cell sensitivity to TAM as compared with vector control cells (Fig. 2b).

To further obtain the insight into the role of linc-RoR in estrogen-independent growth and tamoxifen resistance, we performed a rescue experiment, that is, re-expression of linc-RoR in KO cells. As expected, estrogen deprivation- and tamoxifen-mediated cell growth inhibition was alleviated by linc-RoR re-expression in KO cells (Fig. 2c and d), further supporting a critical role of linc-RoR in estrogen-independent growth.

### Linc-RoR confers ligand-independent activation of ER signaling

**Fig. 3:**
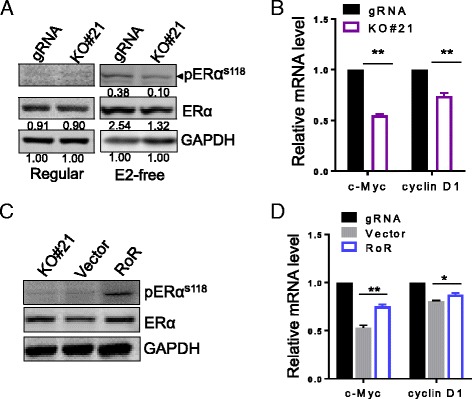
Linc-RoR KO abrogates ligand-independent activation of ER. **a** Linc-RoR KO suppresses estrogen deprivation-induced activation of ER. The intensity value is relative to GAPDH as 1. **b** Linc-RoR KO causes a significant reduction of ER non-genomic target genes mRNA level under E2-free condition. **c** Re-expression of linc-RoR restores activation of ER. **d** Linc-RoR is required for ER non-genomic target gene expression. Re-expression of linc-RoR confirms the capability of estrogen-independent activation, n = 3, *P < 0.05, **P < 0.01

In contrast to the relatively well-characterized estrogen-dependent activation of transcription, ERα can be activated in absence of cognate hormone by processes referred to as ligand-independent activation [28]. In this case, phosphorylation at Ser118 facilitates ligand-independent activation of ER, and thus confers endocrine resistance [29, 30]. We thus sought to test the possibility that linc-RoR might have any role in ligand-independent activation of ER at Ser118. First, we examined the phosphorylation of ER at Ser118 in gRNA control and linc-RoR KO cells under regular and E2-free condition. Not surprisingly, knockout of linc-RoR dramatically suppresses the activation of ER under E2-free condition, but had no effect on phosphorylation of ER under regular condition (Fig. 3a). We next analyzed the expression of c-Myc and cyclin D1, the two non-genomic ER target genes that are upregulated by E2-free treatment (Fig. 1b), in gRNA and linc-RoR KO cells after E2-free treatment. As shown in Fig. 3b, linc-RoR KO significantly decreased the expression of those two non-genomic target genes. These results suggest that linc-RoR KO abrogates estrogen-independent activation of ER signaling. In addition, rescue experiment further confirmed the role of linc-RoR in ligand-independent activation of ER. Western blot also indicated that phosphorylation of ER was restored by linc-RoR (Fig. 3c); consistent with these findings, we also found that re-expression of linc-RoR increased the levels of c-Myc and cyclin D1, as detected by qRT-PCR (Fig. 3d).

### Linc-RoR is required for MAPK/ERK-mediated activation of ER

**Fig. 4:**
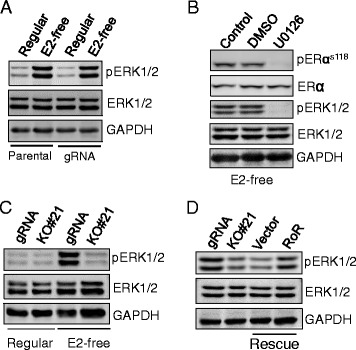
Linc-RoR promotes ligand-independent activation of ER via MAPK/ERK pathway. **a** ERK is activated after 6 days estrogen deprivation. **b** U0126, the MEK inhibitor, abolishes the activation of ERK and ER in response to estrogen deprivation. gRNA control cells were cultured in E2-free medium for 6 days, and then cells were treated with 20 μM U0126 by 8 h before harvesting for Western blot. **c** Linc-RoR KO suppresses the activation of ERK induced by estrogen deprivation. **d** Re-expression of linc-RoR restores the phenotype of ERK activation

It is known that activation of the MAPK/ERK signaling cascade pathway can promote phosphorylation of ER at Ser118 [31, 32]. Thus, we asked whether linc-RoR contributes to estrogen-independent activation of ER through MAPK/ERK signaling pathway. First, we studied the response of ERK to estrogen deprivation in parental and gRNA control cells by Western blot. As shown in Fig. 4a, estrogen deprivation remarkably activated ERK. By contrast, inhibition of ERK by U0126 abrogated estrogen deprivation-induced ERK phosphorylation and activation of ER (Fig. 4b), suggesting the role of MAPK/ERK in estrogen-independent activation of ER signaling. Next, we tested the role of linc-RoR in estrogen deprivation-induced activation of MAPK/ERK pathway. The induction of ERK by estrogen deprivation was significantly repressed in linc-RoR KO cells (Fig. 4c and Additional file 1: Figure S1B). Rescue experiments further confirmed that re-expression of linc-RoR restored ERK activation in E2-free medium (Fig. 4d). Together, these results suggest that linc-RoR plays a role in estrogen deprivation-induced activation of ER via MAPK/ERK pathway.

### Linc-RoR is required for the stability of ERK phosphatase DUSP7

**Fig. 5:**
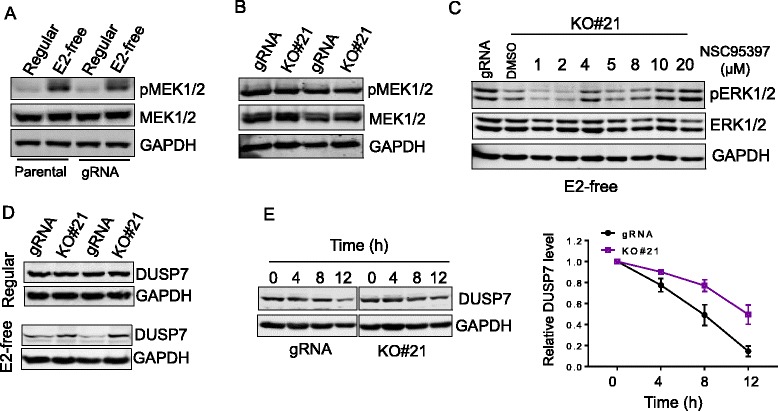
Linc-RoR activates ERK by promoting the degradation of DUSP7. **a** MEK is activated in response to estrogen deprivation. **b** Detection of MEK activity in gRNA and RoR KO cells in E2-free condition. Note that there was no difference in MEK activity between gRNA and RoR KO. **c** Suppression of MKP activity by NSC97397 restores ERK activation in linc-RoR KO cells under E2-free condition. **d** Linc-RoR KO prevents DUSP7 from dagradation under E2-free conditon. **e** Linc-RoR KO increases protein stability of DUSP7. MCF-7 gRNA and linc-RoR KO cells were treated with CHX (cycloheximide) at 20 μg/ml and then were harvested for western blot at indicated time points. Half-life curve is on the right

Given that ERK activity is activated by the upstream kinase MEK, we determined the expression and activation of MEK1/2 in response to estrogen deprivation. As expected, the induction of MEK1/2 was detected after estrogen deprivation in parental MCF-7 and vector control cells (Fig. 5a). We then examined the activation of MEK1/2 in response to estrogen deprivation in gRNA and KO cells. Surprisingly, the difference of MEK1/2 activation was not detected between gRNA and linc-RoR KO cells (Fig. 5b), suggesting that additional mechanism might be involved in linc-RoR-mediated ERK activation by estrogen deprivation.

It is well known that MAPK/ERK signaling is negatively regulated by mitogen-activated protein kinase phosphatases (MKPs). Thus, we employed MKP inhibitor NSC95397 to study the possible involvement of MKPs in this apsect. Linc-RoR KO cells were cultured in E2-free medium for 6 days, and then different concentrations of NSC95397 (1, 2, 4, 5, 8, 10, 20 μM) treated for 3 h before harvesting for Western blot. The dramatic restoration of ERK activation was observed in linc-RoR KO cells at 10 and 20 μM (Fig. 5c). The dramatic upregulation of ERK phosphorylation was also observed in gRNA control cells (Additional file 1: Figure S1C). These results indicate that MKPs play roles in the requirement for linc-RoR in estrogen deprivation-induced ERK activation. MKP family consist of six distinct groups based on their physiological functions; among them, groups 1 ~ 4 are involved in dephosphorylation of ERK [33]. Thus, we selected one member each from these four groups and they were DUSP1/MKP-1, DUSP4/MKP-2, DUSP6/MKP-3 and DUSP7/MKP-X. The expression of MKP-1 was detected in MCF-7 cells under regular condition, and there was a decrease of MKP-1 after E2-free treatment (Additional file 1: Figure S2A and B). However, there was no difference in the MKP-1 level between gRNA and linc-RoR in regular or E2-free condition (Additional file 1: Figure S2A and B). We also examined the expression of MKP-2 and MKP-3; none of them were detectable in MCF-7 cells in regular or E2-free condition (Additional file 1: Figure S2C and D). Finally, we identified DUSP7/MKP-X as a potential candidate. For instance, DUSP7/MKP-X was detected both in gRNA and linc-RoR KO cells under regular condition. Furthermore, there was a decrease of DUSP7 in both gRNA control or linc-RoR KO cells after E2-free treatment. More importantly, the expression of DUSP7 in gRNA was lower than in linc-RoR KO cells (Fig. 5d). These results suggest that DUSP7 might be involved in linc-RoR-mediated ERK activation in response to estrogen deprivation.

To better understand the role of linc-RoR in DUSP7 expression, we examined the half-life of DUSP7. After addition of protein synthesis inhibitor cycloheximide (CHX), we found that DUSP7 degraded much faster in gRNA cells than in linc-RoR KO cells (Fig. 5e). For instance, the half-life of DUSP7 was ~8 h in gRNA cells, whereas it was more than 12 h in linc-RoR KO cells. Together, these results suggest that linc-RoR promotes ERK activation by facilitating DUSP7 degradation.

### DUSP7 is down-regulated in ER+ breast cancer and associated with patient outcomes

**Fig. 6:**
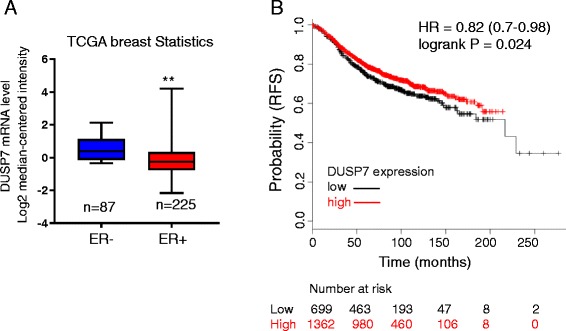
The clinical relevance of DUSP7 in breast cancer. **a** Oncomine data shows DUSP7 mRNA expression in ER negative (ER-) breast cancer tissue (*n* = 87) vs ER positive (ER+) breast cancer tissue (*n* = 225), **P < 0.01. **b** Kaplan-Meier survival curves suggests poor relapse-free survival (RFS) with low expression of DUSP7 compared with those in the high DUSP7 expression group

To determine the clinical relevance of DUSP7 in breast cancer, we interrogated the TCGA breast cancer database at Oncomine and found that DUSP7 was lower in ER+ breast cancer samples (*n* = 225) than in ER- samples (*n* = 87) (Fig. 6a). Importantly, Kaplan-Meier plotter analysis indicated that the low level of DUSP7 was associated with poor RFS among ER+ patients (Fig. 6b). For example, the upper quartile survival of low DUSP7 expression cohort is 60 months, whereas the upper quartile survival of high DUSP7 expression cohort is 78.48 month. Together, these results highlight the clinical significance of DUSP7 in breast cancer.

## Discussion

Estrogen-independent activation of ER through MAPK/ERK pathway was reported previously and its role in endocrine therapies resistance has been well documented, however, the precise underlying mechanism of its regulation is still largely unknown. In this study, we identified and characterized linc-RoR as a novel regulator of MAPK/ERK signaling cascade and thereby may have critical roles in ligand-independent growth of breast cancer cells. Several lines of evidence support this notion. First, linc-RoR is induced by estrogen deprivation; linc-RoR KO results in suppression of cell growth under estrogen deprivation and increases the sensitivity of MCF-7 cells to tamoxifen. Second, linc-RoR KO abolishes the activation of ER and its downstream target under estrogen deprivation. Third, linc-RoR activates the MAPK/ERK cascade upon estrogen deprivation, whereas this activation is dramatically repressed in linc-RoR KO cells. Forth, re-expression of linc-RoR in KO cells restores the estrogen deprivation-induced activation of ER and ERK. Fifth, as an important regulator of ERK, DUSP7 is less stable (shorter half-life) in linc-RoR KO cells than in vector control cells.

ER is the primary modular protein responsible for most effects of estrogen on breast cancer cells. The classical model of ER activation involves the binding of estrogen to ER; on the other hand, ER can also be activated in ligand-independent manner. It is well known that phosphorylation of ER is important for its estrogen-independent activation and endocrine therapy response in breast cancer. The serine118 residue of ER is a well-studied site which is known to be activated by MAPK/ERK in response to estrogen deprivation and thus facilitates the interaction of ER and coactivators, leading to the ligand-independent activation of ER and resistance to endocrine therapy [34, 35]. Work in many laboratories has reported the phosphorylation of ER at Ser118 is upregulated in tamoxifen-resistant MCF-7 cells [36, 37]. Importantly, low level of phosphorylation of ER at Ser118 also is associated with significantly improved disease-free and overall survival [38]. In this study, we demonstrate that E2-free treatment induces the activation of ER at Ser118 and results in the upregulation of ER target genes. However, both ER activation and upregulation of its target gene are abrogated by linc-RoR KO. Importantly, re-expression of linc-RoR in KO cells substantially restores the activation of ER as well as its target gene expression under estrogen deprivation. These findings suggest that linc-RoR might be required for estrogen-independent activation of ER.

Mitogen-activated protein kinase (MAPK) pathways are evolutionarily conserved kinase modules involved in the regulation of fundamental cellular processes such as cell growth, proliferation, survival, migration and apoptosis [39, 40]. A number of studies have hinted that the aberrant activated MAPK/ERK cascade plays a critical role in many aspects of tumorigenesis. Nearly 50% of human malignancies exhibit unregulated ERK signaling [41]. In breast cancer, the upregulated MAPK/ERK signaling is correlated with poor survival in triple-negative breast cancer patients [42]. In addition, the MAPK/ERK pathway also influences chemotherapeutic drug resistance to doxorubicin and paclitaxel in breast cancer cells [43]. In consistence with these results, our analysis of ERK and ER activation also suggests that the MAPK/ERK signaling cascade is activated upon estrogen deprivation, leading phosphorylation of ER at Ser118. ERK inhibitor U0126 dramatically alleviates estrogen deprivation-induced ER phosphorylation at Ser118, suggesting that the estrogen deprivation-induced ER activation is dependent on ERK activation.

The role of lncRNAs MAPK/ERK pathway has been reported in literature. Li et al. showed that lncRNA CASC2 suppresses proliferation of gastric cancer cells by inactivating ERK and JNK [44]. Moreover, lncRNA MALAT1 is able to promote the proliferation and metastasis of gallbladder cancer cells by activating the ERK/MAPK pathway [45]. However, up to date there is no evidence that any lncRNA is involved in estrogen-independent activation of MAPK/ERK signaling. We show that although there is no difference in the phosphorylation of ERK between gRNA control and linc-RoR KO cells under regular condition, the activation of ERK is considerably lower in linc-RoR KO than in gRNA control cells under E2-free condition. In addition, re-expression of linc-RoR in KO cells reverses the phosphorylation of ERK. This result highlights the critical role of linc-RoR in estrogen deprivation-induced MAPK/ERK pathway activation.

It is well known that ERK is positively regulated by MEK and negatively regulated by MKPs. Since there is no difference in MEK activation between gRNA and linc-RoR KO cells either in regular or E2-free condition, we explore the possible involvement of MKPs in linc-RoR-mediated ERK activation in response to estrogen deprivation because the loss of MKPs has been shown to cause the constitutive activation of ERK signal pathway [46–48]. In support of these findings, we show that the downregulation of ERK activation in linc-RoR KO cells is restored by MKP inhibitor NSC95397 under E2-free condition. In particular, DUSP7 appears to play a critical role estrogen deprivation-induced ERK activation in MCF-7 cells. Despite a report that high expression of DUSP7 may play a role in development of leukemia [49], other studies also suggest that a low level of DUSP7 is associated with progression of kidney cancer [50]. Here we provide evidence that DUSP7 is downregulated in response to estrogen deprivation and linc-RoR KO significantly increases the half-life of DUSP7, possibly resulting in the inactivation of ERK. Since p53 can induce DUSP7 [51] and linc-RoR can strongly suppress p53 translation [17], it is our speculation that linc-RoR/p53 axis may play a role in regulation of ERK phosphorylation. Nevertheless, the detailed mechanism remains to be determined yet.

Finally, the clinical data analysis also supports the significance of DUSP7 in breast cancer. For instance, DUSP7 level is lower in ER+ breast tumors than in ER- breast tumors. Particularly, the low expression of DUSP7 is correlated with poor prognosis (Fig. 6b). Therefore, it would be interesting to determine in future whether the linc-RoR/DUSP7/ERK axis may serve a target for therapeutic intervention.

## Conclusions

**Fig. 7:**
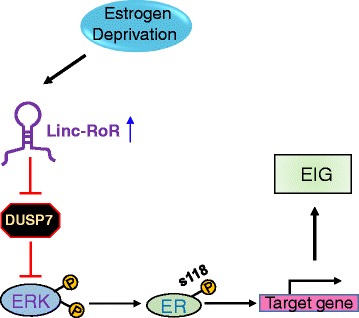
The working model of present study. Linc-RoR promotes estrogen-independent growth (EIG) of ER+ breast cells through conferring the activation of MAPK/ERK pathway. See text for explanation

In summary, our results highlight the novel functional impact of linc-RoR in promoting estrogen-independent growth of ER+ breast cancer. In response to estrogen deprivation, linc-RoR is induced and protects ERK from dephosphorylation via promoting degradation of DUSP7. The activated ERK in turn phosphorylates ER at Ser118 residue and thus promoters the ligand-independent activation of ER, resulting in estrogen-independent growth of breast cancer cells (Fig. 7).

## Additional file

Additional file 1: Table S1. Primers used in this study. **Figure S1.** Expression of linc-RoR and ERK in response to estrogen deprivation. (A) Linc-RoR is induced by estrogen deprivation, whereas this induction is abrogated by addition of E2. Estrogen deprivation: MCF-7 cells were cultured for 24 h in E2-free medium. (B) Linc-RoR is required for the activation of ERK by estrogen deprivation. (C) Suppression of MKP activity by NSC97397 restores ERK activation both in gRNA and linc-RoR KO cells under E2-free condition. **Figure S2.** Expression of MKP1 ~ 3 in MCF-7 cells. (A) Expression of MKP-1 in MCF-7 cells under regular and E2-free condition. (B) MKP-2 is not detectable in MCF-7 cells in either regular or E2-free medium. MDA-MB-231 cell lysate serves as a positive control per the vendor’s datasheet. (C) MKP-3 is not detectable in MCF-7 cells in either regular or E2-free medium. HepG2 cell lysate serves as a positive control per the vendor’s datasheet. (PDF 954 kb)

## Abbreviations

DUSP7: Dual Specificity Phosphatase 7
E2: 17β-estradiol
ED: estrogen deprivation
ER: estrogen receptor
ERK: extracellular signal-regulated kinase
KO: knockout
linc-RoR: lincRNA regulator of reprogramming
lncRNA: long non-coding RNA
MAPK: mitogen-activated protein kinase
MKP: mitogen-activated protein kinase phosphatase
RFS: relapse free survival
TAM: 4-hydroxy tamoxifen
