# Supplementary material for: Linc-RoR promotes MAPK/ERK signaling and confers estrogen-independent growth of breast cancer
Source: Mol Cancer. 2017 Oct 17;16:161. doi: 10.1186/s12943-017-0727-3 (PMC5645922; doi:10.1186/s12943-017-0727-3)
Supplement: Additional file 1: Table S1. — Primers used in this study. Figure S1. Expression of linc-RoR and ERK in response to estrogen deprivation. (A) Linc-RoR is induced by estrogen deprivation, whereas this induction is abrogated by addition of E2. Estrogen deprivation: MCF-7 cells were cultured for 24 h in E2-free medium. (B) Linc-RoR is required for the activation of ERK by estrogen deprivation. (C) Suppression of MKP activity by NSC97397 restores ERK activation both in gRNA and linc-RoR KO cells under E2-free condition. Figure S2. Expression of MKP1 ~ 3 in MCF-7 cells. (A) Expression of MKP-1 in MCF-7 cells under regular and E2-free condition. (B) MKP-2 is not detectable in MCF-7 cells in either regular or E2-free medium. MDA-MB-231 cell lysate serves as a positive control per the vendor’s datasheet. (C) MKP-3 is not detectable in MCF-7 cells in either regular or E2-free medium. HepG2 cell lysate serves as a positive control per the vendor’s datasheet. (PDF 954 kb) [file 12943_2017_727_MOESM1_ESM.pdf]

Table S1. Primers used in this study

Linc-RoR-RT-5.1A	TATAATGAGATACCACCTTA
Linc-RoR-RT-3.1A	AGGAACTGTCATACCGTTTC
c-Myc-RT-5.1	CCTACCCTCTCAACGACAGC
c-Myc-RT-3.1	CTCTGACCTTTTGCCAGGAG
pS2-RT-5.1	ttgtggttttcctggtgtca
pS2-RT-3.1	ccgagctctgggactaatca
CXCL12-RT-5.1	tgagagctcgctttgagtga
CXCL12-RT-3.1	ggaaatgtcaccttgccaac
Cyclin D1-RT-5.1	ATGGAACACCAGCTCCTGTG
Cyclin D1-RT-3.1	ACCTCCAGCATCCAGGTGGC

Figure S1

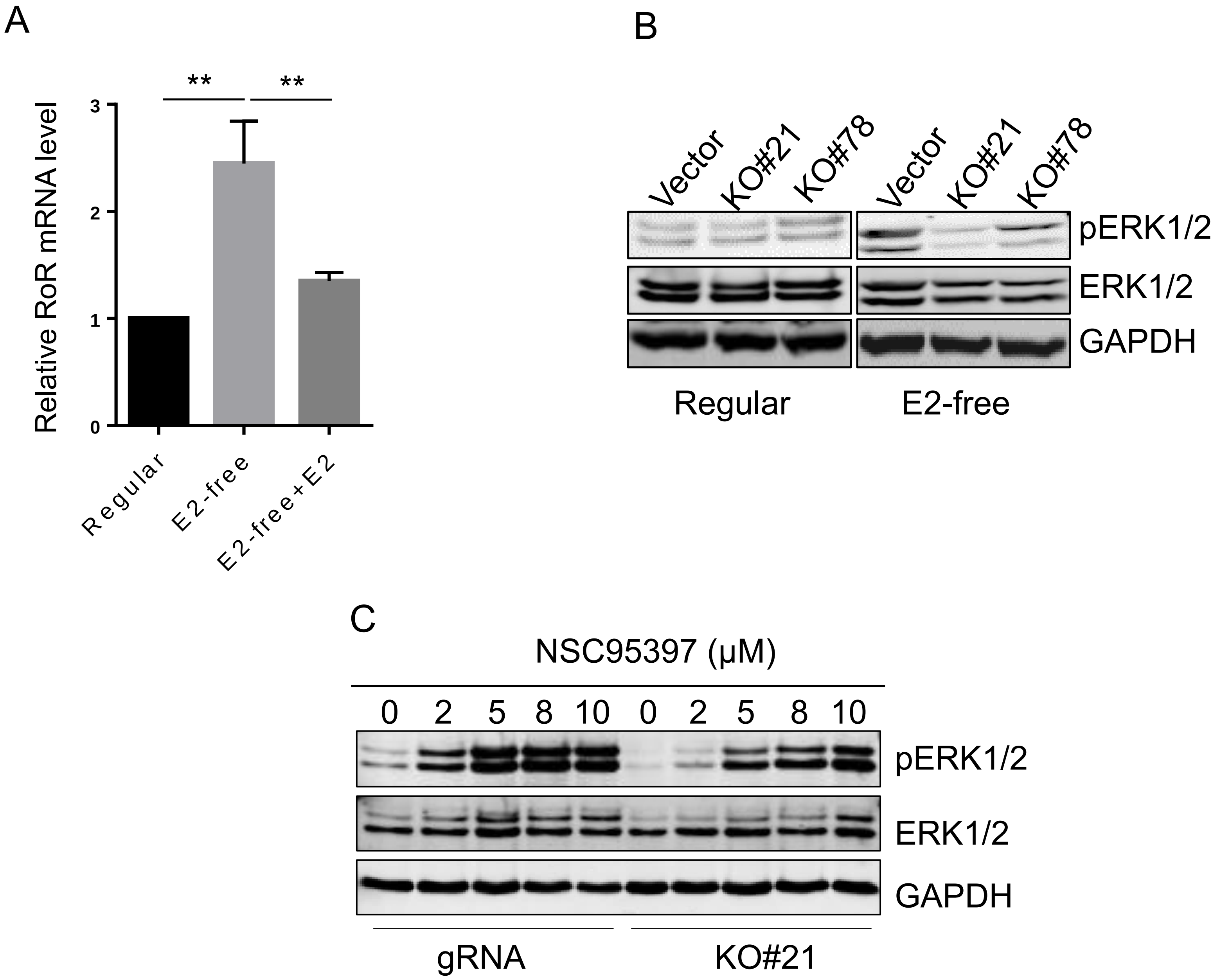
A
Relative RoR mRNA level
0
1
2
3
**
**
Regular
E2-free
E2-free+E2
B
Vector
KO#21
KO#78
Vector
KO#21
KO#78
pERK1/2
ERK1/2
GAPDH
Regular
E2-free
C
NSC95397 (μM)
0
2
5
8
10
0
2
5
8
10
pERK1/2
ERK1/2
GAPDH
gRNA
KO#21

Figure S2

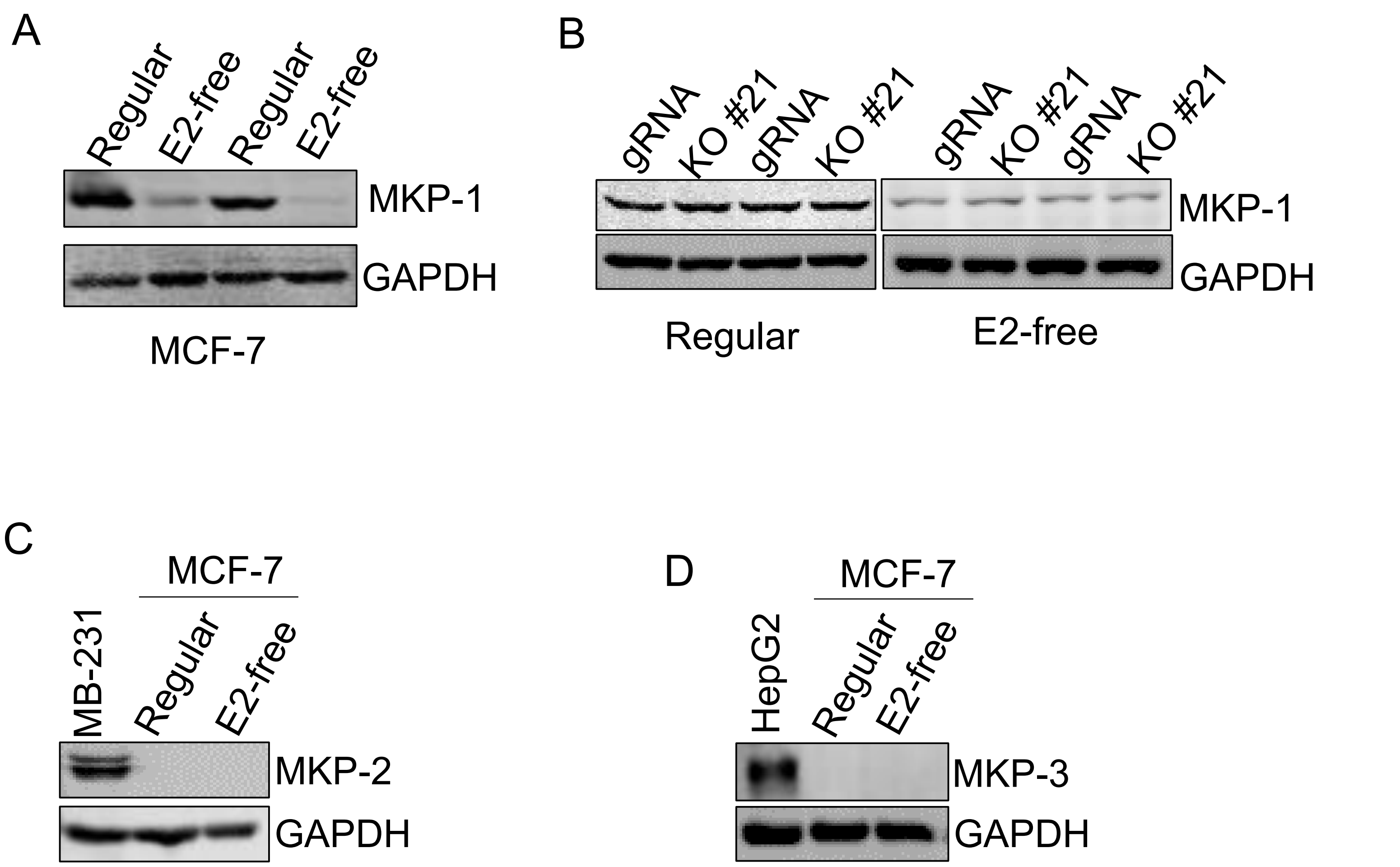
A
Regular
E2-free
Regular
E2-free
MKP-1
GAPDH
MCF-7
B
gRNA
KO #21
gRNA
KO #21
gRNA
KO #21
gRNA
KO #21
MKP-1
GAPDH
Regular
E2-free
C
MCF-7
MB-231
Regular
E2-free
MKP-2
GAPDH
D
MCF-7
HepG2
Regular
E2-free
MKP-3
GAPDH

Figure S1. Expression of linc-RoR and ERK in response to estrogen deprivation. (A) Linc-RoR is induced by estrogen deprivation, whereas this induction is abrogated by addition of E2. Estrogen deprivation: MCF-7 cells were cultured for 24 h in E2-free medium. (B) Linc-RoR is required for the activation of ERK by estrogen deprivation. (C) Suppression of MKP activity by NSC97397 restores ERK activation both in gRNA and linc-RoR KO cells under E2-free condition.

Figure S2. Expression of MKP1~3 in MCF-7 cells. (A) Expression of MKP-1 in MCF-7 cells under regular and E2-free condition. (B) MKP-2 is not detectable in MCF-7 cells in either regular or E2-free medium. MDA-MB-231 cell lysate serves as a positive control per the vendor's datasheet. (C) MKP-3 is not detectable in MCF-7 cells in either regular or E2-free medium. HepG2 cell lysate serves as a positive control per the vendor's datasheet.
